# Pre- and Postnatal Determinants Shaping the Microbiome of the Newborn in the Opinion of Pregnant Women from Silesia (Poland)

**DOI:** 10.3390/life13061383

**Published:** 2023-06-13

**Authors:** Karolina Krupa-Kotara, Mateusz Grajek, Martina Grot, Martina Czarnota, Agata Wypych-Ślusarska, Klaudia Oleksiuk, Joanna Głogowska-Ligus, Jerzy Słowiński

**Affiliations:** 1 Department of Epidemiology, Faculty of Public Health in Bytom, Medical University of Silesia in Katowice, 41-902 Bytom, Poland; awypych@sum.edu.pl (A.W.-Ś.); koleksiuk@sum.edu.pl (K.O.); jglogowska@sum.edu.pl (J.G.-L.); jslowinski@sum.edu.pl (J.S.); 2 Department of Public Health, Department of Public Health Policy, Faculty of Public Health in Bytom, Medical University of Silesia in Katowice, 41-902 Bytom, Poland; mgrajek@sum.edu.pl; 3 Student Scientific Society, Department of Epidemiology, Faculty of Public Health in Bytom, Medical University of Silesia in Katowice, 41-902 Bytom, Poland; d201137@365.sum.edu.pl (M.G.); martinaczarnota@op.pl (M.C.); 4 Doctoral School, Medical University of Silesia in Katowice, 40-055 Katowice, Poland

**Keywords:** microbiome, prenatal factors, fetus, newborn, intrauterine environment, pregnancy, knowledge

## Abstract

Pre- and postnatal factors influence the formation of the newborn’s microbiome as early as birth and the intrauterine period has a substantial impact on the composition of the baby’s gastrointestinal microbiota and its subsequent development. This study intends to measure pregnant women’s knowledge of the importance of microbiota for the health of the newborn. The sample was selected based on defined inclusion and exclusion criteria. The assessment of women’s knowledge was assessed by the Kolmogorov–Smirnov and Kruskal–Wallis statistical tests. This study population comprised 291 adult pregnant women with a mean age of 28.4 ± 4.7 years. A total of 34% (n = 99), 35% (n = 101), and 31.3% (n = 91) were at the 1–3 trimester, respectively. The results showed that 36.4% of the women were aware that the intrauterine period changes the makeup of the gastrointestinal microbiota, whereas 5.8% exhibited awareness of the composition of the child’s normal gut microbiota. Most of the women surveyed—(72.1%)—know that colonization of the tract occurs as early as the birth period. Women with student status (those who will pursue higher education in the future) and those who had given birth to the most children exhibited higher levels of knowledge.

## 1. Introduction

The microbiota performs many vital functions, including activating intestinal angiogenesis, and supporting the maturation and growth of the intestinal epithelial barrier, as well as performing metabolic functions, which include fermentation and the breakdown of undigested food residues, production of B vitamins, vitamin K, and the production of short-chain fatty acids. Depending on the area of the human digestive tract section, a very large number of microorganisms reside in it, ranging from 10^6^ to 10^12^ in 1 g of content. These microorganisms even exceed the number of cells in the human body, and the number of genes of these microorganisms is even 100 times greater than the number of genes in humans [1,2]. Different chemical and physical factors interact in different sections of the gastrointestinal tract, so the microbiota in the oral cavity, stomach and intestines is diverse [3,4].

The proper colonization of the gastrointestinal tract with microorganisms is important to maintain the body’s homeostasis. The intestinal microbiota influences the maturation and development of lymphoid tissues in the gastrointestinal tract, the maturation of epithelium in the intestines, maintains, and strengthens the continuity of the mucosa and activates intestinal immune defenses. Scientific reports indicate a significant effect on the mothers’ knowledge of probiotics on age, education, and use of breastfeeding methods [5,6,7]. 

Pre- and postnatal factors that affect the colonization of the gastrointestinal tract include the type of birth, gestational age, feeding method, and exposure to antibiotics. The reference microbiota is the microbiota of a term-born newborn because of natural childbirth and of being fed with breast milk. These factors can correctly determine the development of the immune system and reduce the risk of infection along the gut–brain axis track [8,9,10,11,12,13]. The predominant part of the microbiota in a healthy newborn is made up of microorganisms of the genus *Bifidobacterium* and *Lactobacillus*. A physiologically healthy fetus in the mother’s uterus has a sterile gastrointestinal tract, thanks to the presence of pus that separates the sterile uterine cavity from the bacteria-populated vagina [14,15]. In addition, bactericidal substances such as lactoferrin and defensin are released into the fetal waters [16,17]. The composition of the gastrointestinal microbiota in the newborn is characterized by high variability during ontogenetic development. Bacteria appear already in the fetal life, constituting the sterile period and their presence has been observed in the placenta and amniotic fluid. However, it is the mother’s gut and vaginal microbiota that are the primary sources of the newborn’s microbiome [18,19,20,21].

The colonization of the gastrointestinal tract of the newborn already begins at the time of delivery; this is the first and main factor, so the type of bacteria is important. The route of delivery affects the number and type of bacteria that are acquired by the newborn [22]. According to WHO recommendations, only 10% to 15% of births should end in cesarean section. Yet, all over the world, including Poland, a significant increase in pregnancies ending in this type of delivery has been observed in recent years. Increasingly, the only indication for obstetric surgery is the individual feelings of women, which has an impact on the increased number of cesarean sections performed “on request” [23,24]. It should be noted that this type of birth is not natural; in the newborn, in the first minute of life, the conditions for normal respiratory function and adaptation of the cardiovascular system are not met. It is these newborns who are more prone to circulatory and respiratory disorders than children from natural childbirth [25]. 

During natural childbirth, the newborn encounters bacteria in the mother’s vagina. Within the first 24 h after birth, the baby’s gastrointestinal tract is colonized by *Staphylococcus*, *Lactobacillus*, and *Enterococcus* bacteria; these bacteria are responsible for activating immune cells and preparing the environment in the intestines for the growth of anaerobic bacteria [26]. When a baby is born by cesarean section, it is deprived of contact with the bacteria found in the mother’s vagina. Its digestive tract becomes colonized by bacteria found on the skin of the mother and/or medical personnel, and those found in the hospital environment. It primarily contains bacteria of the genera *Corynebacterium*, *Propionibacterium*, *Staphylococcus,* and *Clostridium*. The microbiota of cesarean-section babies has a reduced number of *Bifidobacterium,* once *Bacteroides*, and colonization of their digestive tract occurs more slowly than in babies born during natural delivery [27]. In addition, delivery by cesarean section delays skin-to-skin contact with the mother and rooming-in of mother and baby. All these factors delay breastfeeding and promote the colonization of bacteria from the hospital environment. Colonization of the gastrointestinal tract by unfavorable bacteria can lead to damage to the intestinal mucosa, stimulate an inflammatory response, and lead to the development of autoimmune and allergic diseases [28]. Studies have shown that cesarean sections may be associated with the occurrence of immune-mediated diseases, allergies, or type 1 diabetes in children. It has also been observed that diarrhea, atopic dermatitis, allergic rhinitis, and asthma are more common in the first year of life [29,30,31]. 

Another factor that affects the colonization of the digestive system is the method of feeding. Breast milk can be described as a natural symbiotic, as it contains prebiotics as well as probiotics, especially oligosaccharides, which increase the growth in beneficial bacteria. Breastfed babies have an increase in *Bifidobacterium*, which are responsible for colonizing the digestive tract. In early milk, the concentration of oligosaccharides is 1.2–2.3 g/100 mL, while in mature milk it is 0.8–1.2 g/100 mL [32,33]. The fermentation products of these bacteria protect the baby’s digestive system from pathogenic strains of *Clostridium perfringe* and *Escherichia coli*. Colostrum plays a special role. In its composition, it contains bacteria of the genus *Bifidobacterium* and *Lactobacillus,* and oligosaccharides, which stimulate the growth of these bacteria [34]. Feeding colostrum results in better colonization of the intestinal microbiota and the formation of the immune system. Delayed colostrum secretion after a cesarean section and postponement of breastfeeding adversely affects the newborn’s microbiome. The microbiome of artificially fed newborns is more diverse but contains up to 10 times less lactic fermentation bacteria [35]. Milk replacers, on the other hand, cause the proliferation of bacteria from the *Enterobacterium* and *Bacteroides families* [36,37,38,39]. To promote healthy newborn microbiota, mothers should be aware of the benefits of normal birth and breastfeeding practices. Therefore, this study was conducted to assess pregnant women’s knowledge of the importance of microbiota for newborn health.

In addition, we aimed to assess (1) the relationship between maternal sociodemographic factors and women’s knowledge of the importance of the neonatal microbiota; an (2) the impact of information sources on the mothers’ knowledge of the neonatal microbiota in the context of women’s knowledge in this area of science.

## 2. Materials and Methods

### 2.1. Description of this Study’s Design

This study initially recruited 312 women. After reviewing the questionnaires, 21 participants were excluded from this study due to incomplete responses or failure to meet the inclusion criteria described below. Finally, 291 female respondents between the ages of 18 and 41 were eligible for this study. 

This study included 291 women, with an average age of 28.4 ± 4.7 years. The characteristics of this study’s patients are shown in Table 1. 

### 2.2. Eligibility Criteria 

The sample was selected based on the following criteria: the physical and cognitive ability to provide voluntary consent to take part in this study, being admitted or receiving prenatal care at two institutions in the Department of Gynecology and Obstetrics, Pregnancy Pathology, and Gynecologic Endocrinology with Gynecologic Oncology and Endoscopic Endometriosis Treatment Subdivision at the Center for Women’s and Children’s Health of the Specialized Hospital in Zabrze resident in the Silesian province (Poland), and being 18 years of age or older. Patients not meeting the above criteria were not qualified for this study. 

### 2.3. Sampling Strategy

Pregnant women admitted to the department were invited to participate in this study by a member of the research team collecting an anonymous patient database. Patients were briefed about this study’s procedures and asked to sign an informed consent prior to data collection. An interview using a survey questionnaire designed by members of the research team was conducted with each patient individually in the office after a medical consultation (with the relevant specialist). Respondents were not required to complete the questionnaire in its entirety. 

### 2.4. Number of Eligible Women Participants

The minimum required sample size was calculated, based on the size of the population of the Silesian region (Poland). It was estimated that a sample of 291 pregnant women would be sufficient and representative of the Silesian region in Poland. It was assumed, according to the Central Statistical Office (CSO) report, that the population of pregnant women was 34,736 thousand women. The sample size was calculated according to the formula: N_min_ = NP⋅(α^2^⋅f(1 − f)) ÷ NP⋅e^2^ + α^2^⋅f(1 − f), where: N_min_—minimum sample size; NP—the size of the population from which the sample is drawn; α—confidence level for the results; f—the size of the fraction; and e—assumed maximum error. For the population of Silesian pregnant women (Poland), the minimum sample size of respondents was calculated, which was 138 (α = 0.95; f = 0.9; e = 0.05). Th non-normality of the distribution was assessed by the absence of differences between subgroups (*p* > 0.05).

### 2.5. Data Collection Tool

The anonymous, proprietary survey questionnaire (Supplementary Materials) was conducted using a direct survey method in the form of an interview (with multiple-choice questions). The author’s questionnaire was not adapted from other research in the field, but was created based on the literature sources of the subject matter undertaken. The content of the questionnaire consisted of 20 questions—some of the closed, single-choice questions were in two parts. The first concerned metrics and the second contained questions of a testing nature to demonstrate the women’s knowledge of the importance of the gastrointestinal microbiota for the state health of the newborn. In assessing the knowledge of the women surveyed, one point was awarded for each correct answer, while zero points were awarded for an incorrect answer. The women surveyed could obtain a minimum of zero (complete lack of knowledge) and a maximum of 18 points (complete knowledge). A pilot study (n = 50) was carried out among this study’s patients.

Before the actual study, the survey questionnaire was validated by making it available twice (retesting) in a pilot study among 30 independent women. An interval of 3 weeks was maintained between measurements. The average calculated Cohen’s Kappa coefficient for both measurements was 0.78, indicating a very good agreement. In addition, Cronbach’s Alpha coefficient was estimated at 0.82, which is accepted as an expected value in scientific research [40].

### 2.6. Statistical Analysis

The independent variables were level of education, e.g., primary, secondary, and tertiary education, (material status (total economic/financial wealth), and health status). On the other hand, the authors defined the concept of “good health” as a state of good physical, mental, and social well-being. In addition, the mother’s level of knowledge was assessed based on their answers to the questions asked. Details of the knowledge assessment are described in the results section. 

Such characteristics of this study’s population, such as education status, occupational status, marital status, place of residence, and number of births, were used as independent variables in the testing.

To answer the specific research hypotheses (the level of knowledge of pregnant women is at a low level in the context of the newborn microbiome), statistical analyses were performed using the IBM SPSS Statistics package. Frequency analyses and analyses of basic descriptive statistics with the Kolmogorov–Smirnov (parametric) and Kruskal–Wallis (non-parametric) tests to analyze the variance in a between-group design were performed using the data verification program SPSS. The statistical testing conducted made it possible to verify the research hypotheses where *p* < 0.05 was considered the level of significance.

### 2.7. Ethical Statement

This study complies with the provisions of the Declaration of Helsinki as amended. This study’s design, considering the Act of 5 December 1996 of the professions of physician and dentist (Journal of Laws of 2011, No. 277, item 1634, as amended), is not a medical experiment and does not require the approval of the Bioethics Committee of the Silesian Medical University in Katowice. All data were encoded with appropriate symbols preventing the identification of patients described in the Personal Data Protection Act of 29 August 1997 (Journal of Laws of 1997, No. 133, item 883). Prior to contacting patients, gatekeeper consent was secured. All facility managers provided consent before data collection.

## 3. Results

The analysis showed that no woman received either the minimum or the maximum number of points. The most common score in the sample was 14 points, which indicates satisfactory but incomplete knowledge. Next, it was examined which questions proved to be the most difficult and which were the easiest for the women surveyed. The most difficult questions included question four (the predominant part of the microbiota in a healthy newborn born on time is bacteria of the genus…), which was answered correctly by only 5.8% of the respondents, and question five (the predominant part of the microbiota in premature babies is bacteria of the genus…), with the proportion of correct answers at 7.9%. On the other hand, the easiest questions included questions 10 (in breast milk there are/are…) and 20 (a significant influence on the microbiota in the gastrointestinal tract is…), with a 75.6% share of correct answers (Table 2).

**Figure 1:**
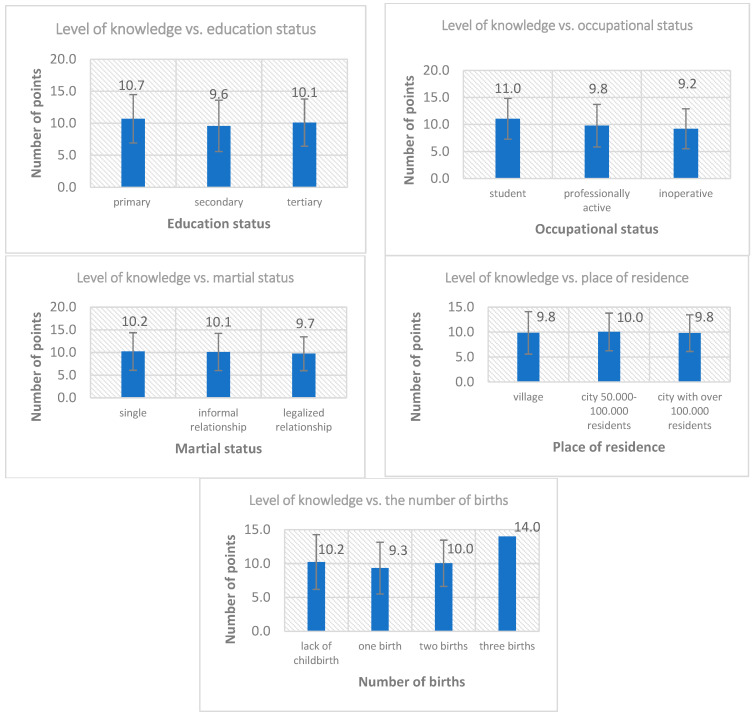
Level of knowledge versus selected variables in the study group of women *. * The whiskers on the box plot show the arithmetic means and standard deviations.

The one-way analysis of variance was performed in a between-group design (Figure 1). However, no results were statistically significant or even close to statistical significance, F (2; 288) = 1.25; *p* = 0.287. The second one-way analysis of variance was performed in a between-group design. Statistically significant results were noted, F (2; 288) = 4.49; *p* = 0.012. Therefore, a post hoc analysis was required. One statistically significant difference was noted. A higher level of knowledge was noted in the group of women who were studying compared to women who were not working (*p* = 0.010). 

Furthermore, there was also a statistical trend difference between female students and working women (*p* = 0.082). Only the differences between those working and not working appeared to have no statistical significance. A third one-way analysis of variance was conducted on an intergroup basis. In the intergroup comparisons, no results were statistically significant or even close to statistical significance, F (2; 288) = 0.38; *p* = 0.687. 

A one-way analysis of variance was then performed in an intergroup design. A result, at the level of a statistical trend, was recorded, F (3; 287) = 2.29; *p* = 0.079. However, such a result did not allow for a post hoc analysis. 

In addition, patients who had three births had a significantly higher level of knowledge and obtained the same results, so there was no variation in the level of knowledge in this group. The 138 respondents who provided this response represented 47.5% of the sample. The response ‘magazines’ was slightly less frequent, indicated by 84 female respondents (28.9%). The two most reliable sources of knowledge, i.e., medical staff and dieticians, appeared much less frequently, with 56 (19.2%) and 13 (4.5%) women, respectively.

## 4. Discussion

A search of medical databases for scientific reports on the level of knowledge about the role of microbiota in shaping the state health of the newborn does not provide a positive answer. It is known that normal gastrointestinal microbiota plays an important role in maintaining the body’s homeostasis through several metabolic functions. The gastrointestinal microbiota are already formed in the newborn at birth, and the intrauterine period has a significant impact on the composition of the child’s gastrointestinal microbiota and its subsequent development [41,42]. Therefore, the study of the level of knowledge and awareness among pregnant women, who are the first line of prevention of their baby’s state of health, is extremely important. 

The survey of women was conducted to assess the state of their knowledge of the importance of the microbiota in the gastrointestinal tract of the newborn, which is extremely important in their further development. A source search of medical databases did not allow us to find other similar studies with which to compare the results obtained. Self-analysis showed that the majority were women aged 26–32, the age group of women who most often decide to start a family and expand it [43].

The microbiota in an organism can be defined as a community of commensal and saprophytic microorganisms residing in the body. They are found both inside the body and on its surface and begin to appear as early as the intrauterine development of the fetus. Only 36.4% of women surveyed are aware that the intrauterine period affects the development of the baby’s digestive system and its subsequent development. Analyses of the placentas of newborns born by cesarean section and by natural delivery showed that in both cases the placentas contained DNA of bacteria that have a positive effect on the immune system of *Bifidobacterium* and *Lactobacillus rhamnosus*. This study confirms the transfer of maternal bacteria to the fetus through the bloodstream [44].

The formation of the gut microbiota is influenced by prenatal and postnatal factors [45,46]. Prenatal includes the transmission of the mother’s microbiome through the bloodstream to the fetus, while postnatal factors consist of the type of delivery, gestational age, and the method of feeding, as many as 84.1% of the women surveyed were able to give the correct answer about the factors that affect the colonization of the gut microbiota in the newborn. Several studies indicate that there is a correlation between the development of certain diseases during the ontogenesis of the newborn’s body and the diversity of the gut microbiome [47,48]. The colonization of the body by microorganisms already occurs during fetal life as many as 72.1% of respondents were able to give the correct answer about at what point the colonization of the baby’s gastrointestinal tract begins. Microorganisms that come from the mother are then found in the umbilical cord, placenta, amniotic fluid, and meconium [49,50]. With the development of medicine, better diagnostic methods have emerged, allowing a more accurate way to study the microorganisms that reside in the human digestive tract and what effect they have on the body. The body’s microbiota consists of the genomes and metabolic products of the microbiota, which determines the proper development of the immune system; for a newborn’s body to function properly in the ectopic environment it must have a properly developed immune system and intestinal barrier [40,41]. In this study, 21% of the women surveyed correctly answered the question that probiotics affect the immune system by increasing local and general immunity, resulting in a lessened impact from harmful bacteria and viruses. 

Premature infants—newborns born 37 weeks before gestation—may develop abnormal gut microbiota that is related to the use of drug treatment, an immune system that has not been fully developed, or long hospitalization. There are some differences in the profile of microorganisms populating the gastrointestinal tract in preterm and term-born newborns. *Enterobacteriaceae* and *Clostridium* predominate in prematurely born infants, while *Bifidobacterium*, *Lactobacillus*, and *Streptococcus* predominate in normal-born infants [51,52]. Only 5.8% of the women surveyed provided the correct answer about the predominant microbiota in newborns born on time and 3% of the women surveyed about the microbiota in premature infants. The predominant bacteria in normal-born newborns are of particular importance because they are among the commonly used probiotics that have a beneficial effect on the maturation of the intestinal epithelium and the immune system. A common disease in premature infants is NEC or necrotizing enterocolitis; abnormal colonization of the intestines by bacteria is considered an important etiological factor. In clinical observations, it has been observed that premature infants fed with breast milk are less likely to develop necrotizing enterocolitis compared to artificially fed premature infants. This is related to the fact that breast milk has properties that strengthen the intestinal immune system and barrier [53,54]. 

The survey shows that 85.2% of women are aware that the best feeding method that affects the development of the gut microbiota is breast milk, and 75.6% of respondents gave the correct answer when asked about the composition of breast milk. Breast milk is the baby’s natural food, and it contains several probiotics including *Lactobacillus* and *Bifidobacterium*, as well as prebiotics, which influence the formation of the infant’s gut microbiota [55]. Some factors can affect the microbiota in breast milk. These can be perinatal factors: duration of pregnancy, type of delivery, lactation period, use of antibiotics during delivery, and maternal factors: diet during pregnancy, BMI, and immunological diseases [56,57]. According to studies, a high-fat diet in pregnant women and during lactation results in an increased colonization of the intestines by *Bacteroides* and subsequent colonization of the newborn with these bacteria [58].

A study that observed children up to the age of seven showed that their gut microbiota composition with normal weight compared to a group of children with a predisposition to overweight changes significantly. In the future, observation of the intestinal microbiota and its changes may provide a tool for early diagnosis, prevention, and introduction of appropriate treatment for overweight and obesity in children [59]. Appropriate therapy with probiotics and prebiotics can be used to maintain a healthy gut microbiome and a fiber-rich diet can be introduced. Studies show that probiotics protect newborns from inflammatory bowel disease, infections, diarrhea, and atopic dermatitis. The use of probiotics in pregnant women can modulate the composition of the bacterial flora in the newborn [60,61]. In addition, the use of prebiotics has a positive effect on the gut microbiome. In a study by Baldassarre et al. [62], it was observed that their supply modulates the microorganisms that settle in the gut of a newborn. It was shown that the use of prebiotics in children between one and six months of age increased the number of positive bacteria, while it decreased the number of *Escherichia coli* [63,64].

In the survey conducted, 37.8% of the women surveyed had a college degree, 52.9% had a high school degree, and 9.3% of the women had primary education. Although it might seem that women with higher education should have a higher level of competence on the topic of the newborn’s microbiota, this study shows that education has no significant effect on women’s awareness of the role played by the intestinal microbiota and how it affects the health of the newborn. Knowledge on the subject can be found in magazines and on the Internet. These are very common sources that everyone has access to and, more than that, the subject is very popular nowadays, but it requires the ability to filter the information obtained.

Comparing the level of knowledge of the women surveyed and their occupational status, a higher level of knowledge was noted in the group of pupils/students (22.7%) compared to non-working women (31.6%) and working women (45.7%). This difference may be because students/graduates are a young age group, showing more interest in newly learned topics that are currently receiving special attention in scientific research, the quality of the body’s microbiota, and the pathological changes that can occur because of its disruption.

This author’s study shows that social status also does not affect the degree of women’s knowledge of the factors that shape the microbiota in the newborn. The survey included 7.6% of women with a maiden status, 30.6% of women in an informal relationship, and 61.9% of women in a formal relationship. Regardless of what status the women surveyed had, it did not affect the answers they gave in the survey. In addition, no correlation was shown between the place of residence and level of knowledge. This may be because access to scientific journals is very widespread and access to the Internet is very easy. Next, it examined whether the number of births differentiates women’s knowledge. 

Given the above, it is important to continue research on the microbiome and implement probiotic therapy for the treatment of various disease entities, as well as to educate the public about leading a lifestyle that promotes a favorable composition of the microbiome. From a future perspective, the correlation between the bioactivity of the microbiota and the bioavailability of functional compounds should be considered, as it is important to modulate the well-being of women and their offspring [64].

The ongoing investigation is important from the point of view of the peculiar fashion of young people, including young mothers to use dietary supplements (including probiotic therapy) on their own. Admittedly, there is no program in Poland to monitor the phenomenon and it is in vain to look for activities aimed at promoting knowledge about the microbiota in the newborn. Given the issues of self-medication, this knowledge should be available to those interested in the topic of intestinal microbiota during this particularly sensitive period of a woman’s life.

Basic knowledge of the microbiota in pregnant women may also be important in communities without high levels of education and easy access to information due to several factors. Firstly, the health of the microbiota is important for good overall health and well-being. Regardless of the level of education or access to information, all people have the right to receive basic health and prevention information. Therefore, education about the microbiota among pregnant women can help improve their health and the health of their future children. Secondly, the impact of the microbiota on the health of the baby can have long-term consequences for the community. If pregnant women are aware of the role of the microbiota in their child’s development and how to look after its health, this could contribute to reducing the risk of certain diseases and conditions in children in the future. This, in turn, can have an impact on reducing healthcare costs and improving the quality of life in communities with limited access to medical services. Education about the microbiota in communities without a high level of education can be delivered in a way that is tailored to the specific characteristics of these communities. Available communication tools and methods can be used, such as graphic educational materials, videos, or group meetings led by professionals. Providing simple, easy-to-understand information about the microbiota, and its impacts on health can be the key to effective education and health promotion in these communities.

## 5. Strengths and Limitations

The undoubted strength of the conducted study is the large group of respondents that were included in the procedure and the fact that the research was conducted by a direct survey method, which is quite unusual in pandemic times, and which ensures the reliability of the conduct of the research project and the obtaining of reliable results. Test power and group size were checked during study planning. In addition, the procedure introduced allowed for a significant minimization of bias. Despite the strengths, this study has limitations that could not be avoided during the planning of the research procedures. The main limitation is the fact that this study was conducted with a proprietary instrument, which may not have exhausted the topic and may not have had all the variables. Thus, it is suggested that further research be conducted on this topic, to expand the current knowledge and research that the authors have undertaken. Given the scientific interest in microbiota, coupled with the increasing availability of probiotic products, it is important for practitioners to understand patient knowledge and opinions about probiotics, as well as their current use in pregnant women and infants, to enable the effective use of scientific knowledge as it becomes available. The research was concentrated only according to the variables of age and socio-demographic status while ignoring many other variables, so further research should be carried out considering the other conditions.

This study also has praxis implications. Given the growing interest in microbiota and the availability of probiotic products, it is important for medical professionals to be knowledgeable about probiotics and their use in pregnant women and newborns. Considering this study’s findings, doctors and other professionals can be more aware of the patients’ knowledge and opinions about probiotics, which will allow them to effectively use the available scientific knowledge. In addition, this study points to the need for further research to expand our knowledge of the determinants of prenatal and postnatal microbiota. This may include consideration of other variables that were missed in this study and a more comprehensive analysis of the relationship between the microbiota and various factors. Further research may contribute to a better understanding of the impact of microbiota on neonatal health and better neonatal care.

In addition, this study suggests that it is important for future research and clinical practice to focus on understanding the patients’ knowledge and opinions about probiotics and their current use in pregnant women and newborns. This can create an informed public that makes informed decisions about probiotic supplementation during the prenatal and postnatal periods. This study focused mainly on age and socio-demographic variables and omitted many other variables. The practical implication is that further research is needed, considering other conditions and factors affecting the microbiota in newborns. This will allow for a more comprehensive analysis and understanding of the processes that shape the microbiota and its impact on children’s health.

## 6. Conclusions

The results of our study allow us to conclude that women’s knowledge is at a satisfactory level. In addition, the analyses carried out showed no correlation between education, social status, and place of residence and the knowledge of the women surveyed regarding the importance of the gastrointestinal microbiota in the newborn. The level of knowledge to a higher degree was possessed, however, by women who were studying and those with the highest number of pregnancies ending in childbirth. The main source of knowledge from which women draw substantive issues is mass media in the form of the Internet.

Promoting knowledge of the gut microbiota in the newborn among young mothers is important, especially in an era of access to dietary supplements, as well as widespread advertising campaigns promoting the use of these products. The consumer, especially the young mother-to-be, should make informed choices to properly influence her child’s health, based on evidence-based medicine and not aggressive marketing.

The key point is that basic knowledge about the microbiota in pregnant women can be important in communities without high levels of education and easy access to information. Such education can contribute to improving the health of women and their babies, reducing the risk of certain diseases, and improving the quality of life in these communities. Therefore, it is worth striving to ensure access to basic knowledge on the subject and to adapt education methods to the specifics of these communities.

## Figures and Tables

**Table 1:** Characteristics of the surveyed female patients.

Variable		n	%
**education**	primary	27	9.3
	secondary	154	52.9
	tertiary	110	37.8
**number of pregnancies**	1	110	37.8
	2	109	37.5
	3	65	22.3
	4	7	2.4
**number of births**	0	129	44.3
	1	114	39.2
	2	45	15.5
	3	3	1.0
**week of pregnancy**	first trimester	99	34.0
	second trimester	101	34.7
	third trimester	91	31.3
**professional status**	pupil/student	66	22.7
	active	133	45.7
	non-working	92	31.6
**marital status**	single	22	7.6
	casual relationship	89	30.6
	formal relationship	180	61.9
**residence**	village	83	28.5
	city of 50–100 thousand residents	108	37.1
	city of more than 100 thousand residents	100	34.4
**material status**	very good	46	15.8
	rather well	184	63.2
	on average	55	18.9
	rather bad	6	2.1
**health status**	very good	54	18.6
	well	164	56.4
	on average	67	23.0
	wrong	6	2.1

**Table 2:** Correctness of answers given by female patients.

Answers	Incorrect		Correct	
	n	%	n	%
Factors that affect the colonization of the gastrointestinal microbiota of the newborn baby	193	66.3	98	33.7
The vast majority of the microbiota of a healthy newborn born on time is bacteria of the genus	274	94.2	17	5.8
The predominant microbiota of preterm infants is bacteria of the genus	268	92.1	23	7.9
Colonization of a newborn’s gastrointestinal tract begins at the time of	81	27.8	210	72.2
A baby who is born by cesarean section is the first to be colonized by bacteria	81	27.8	210	72.2
The best feeding regimen to influence the development of a newborn’s gastrointestinal microbiota is the following	109	37.5	182	62.5
Feeding exclusively with breast milk promotes	84	28.9	207	71.1
In breast milk there are/are	71	24.4	220	75.6
Premature low birth weight babies are at risk of	258	88.7	33	11.3
Intestinal dysbiosis is	157	54	134	46
The microbiota of a child’s digestive system changes with the	84	28.9	207	71.1
The microbiota of the digestive system is fully formed at age	139	47.8	152	52.2
In the immune system of the gastrointestinal tract, probiotics show an important influence on its functions through	159	54.6	132	45.4
In respiratory infections, which include the common cold, probiotics can	86	29.6	205	70.4
Probiotics are effective in infectious diarrhea in children, by	82	28.2	209	71.8
Probiotics play a key role in defending against oral pathogens by	95	32.6	196	67.4
The microbiota of the gastrointestinal tract is significantly influenced by the	71	24.4	220	75.6

## Data Availability

Not applicable.

## Supplementary Materials

The following supporting information can be downloaded at: https://www.mdpi.com/article/10.3390/life13061383/s1.
