# Supplementary material for: Pre- and Postnatal Determinants Shaping the Microbiome of the Newborn in the Opinion of Pregnant Women from Silesia (Poland)
Source: Life (Basel). 2023 Jun 13;13(6):1383. doi: 10.3390/life13061383 (PMC10305644; doi:10.3390/life13061383)
Supplement: Supplementary file 1 [file life-13-01383-s001.zip › life-2370193-supplementary.pdf]

QUESTIONNAIRE

Dear Madam,

We kindly request your permission to participate in this study.

The purpose of the study is to *assess women's awareness of the impact of gut microbiota on the health of the newborn.* The questionnaire is **anonymous** and the results will be used for scientific purposes only.

Please give me honest answers and truthful information, as the value of the research depends on it.

Please mark with an "**X**" the selected answer in the space provided □ or describe in the boxes and do not skip any questions.

The answers are **single-choice**, i.e., we choose only one answer option in for question.

I sincerely thank you for your time.

STATEMENT

I give my informed consent to participate in the study by completing the questionnaire.

- ☐ YES
- ☐ NO

Year of birth ☐

Pregnancy week ☐

Number of pregnancies ☐

Number of births ☐

Height (cm) ☐

Pre-pregnancy body weight (kg) ☐

Current body weight (kg) ☐

Education
- ☐ basic
- ☐ Medium
- ☐ higher

Professional status
- ☐ pupil/student
- ☐ active person
- ☐ non-worker
- ☐ pensioner

Social status
- ☐ Miss
- ☐ in a casual relationship
- ☐ married
- ☐ divorcee
- ☐ widow

Place of residence
- ☐ village
- ☐ city of 50 to 100 thousand inhabitants
- ☐ city of more than 100 thousand inhabitants

How do you assess your material status?
- ☐ very good
- ☐ rather well
- ☐ on average
- ☐ rather bad

How do you assess the state of your health?
- ☐ very good
- ☐ well
- ☐ on average
- ☐ wrong

1. Did you attend a birthing school?
   - ☐ yes
   - ☐ not

2. Where do you get your knowledge about newborn nutrition?
   - ☐ from a doctor
   - ☐ from a nutritionist
   - ☐ from the Internet
   - ☐ from magazines
   - ☐ Other sources (what?) ....

3. Does the intrauterine period have a significant impact on the composition of the baby's gastrointestinal microbiota and its subsequent development?
   - ☐ yes
   - ☐ not
   - ☐ I don't know

4. Factors that affect the colonization of a newborn's gastrointestinal microbiota are:
   - ☐ type of delivery, gestational age, how the newborn is fed
   - ☐ nutrition of a woman during pregnancy
   - ☐ type of delivery, method of feeding the newborn baby
   - ☐ Dietary supplements consumed by a pregnant woman
   - ☐ I don't know

5. The bulk of the microbiota of a healthy newborn born on time is bacteria of the genus:
   - ☐ *Bacteroides*
   - ☐ *Bifidobacteriumi Lactobacillus*
   - ☐ *Enterobacterium*
   - ☐ *Enterobacteriaceae*
   - ☐ I don't know

6. The predominant microbiota of preterm infants is bacteria of the genus:
   - ☐ Clostridium and Eterococcus
   - ☐ Lactobacillus and Bifidobacillus.
   - ☐ Clostridium and Enterobacterius
   - ☐ Staphylococcus
   - ☐ I don't know

7. Colonization of the gastrointestinal tract of a newborn begins at the time:
   - ☐ inseminations
   - ☐ fetal life
   - ☐ delivery
   - ☐ few days after childbirth
   - ☐ I don't know

8. A baby who is born by cesarean section is the first to be colonized by bacteria:
   - ☐ vaginal
   - ☐ found on the mother's skin
   - ☐ located on the skin of the mother or staff
   - ☐ found on the skin of the mother, staff and hospital bacteria
   - ☐ I don't know

9. The best way to feed on the development of a newborn's food microbiota is:

- ☐ feeding with modified milk
- ☐ breast milk feeding
- ☐ I don't know

10. Feeding exclusively with breast milk is conducive:
    - ☐ slower colonization of the gastrointestinal tract of the newborn baby
    - ☐ multiplication of pathogenic bacteria
    - ☐ faster colonization of the gastrointestinal tract of the newborn baby
    - ☐ has no effect on the gastrointestinal tract of the newborn baby
    - ☐ I don't know

11. In breast milk there are/are:
    - ☐ antibodies and other immune components
    - ☐ trans fatty acids
    - ☐ dietary fiber
    - ☐ pathogenic bacteria
    - ☐ I don't know

12. Premature babies with low birth weight are at risk:
    - ☐ type II diabetes
    - ☐ obesity
    - ☐ dysbioses
    - ☐ bacterial proliferative syndrome
    - ☐ I don't know

13. Intestinal dysbiosis is:
    - ☐ qualitative and quantitative disorders in the composition of the gastrointestinal microorganisms
    - ☐ Only qualitative disorders in the composition of gastrointestinal microorganisms
    - ☐ only quantitative abnormalities in the composition of gastrointestinal microorganisms
    - ☐ Excess microorganisms in the digestive tract
    - ☐ I don't know

14. The microbiota of a child's digestive system changes with:
    - ☐ dietary expansion
    - ☐ Expanding the diet and gradually introducing solid foods
    - ☐ does not change
    - ☐ I don't know

15. The microbiota of the digestive system is fully formed at age:
    - ☐ 2-5 years
    - ☐ 7-10 years
    - ☐ 17-20 years
    - ☐ 27-30 years old
    - ☐ I don't know

16. In the immune system of the gastrointestinal tract, probiotics show significant effects on its functions through:
    - ☐ increase local and general immunity, so the impact of bacteria and viruses is reduced
    - ☐ Decrease local immunity and increase general immunity, so the impact of viruses is lower
    - ☐ Lowering local and general immunity, so the impact of bacteria and viruses is reduced

- ☐ Probiotics have no effect on the gastrointestinal immune system
- ☐ I don't know
- ☐

17. In respiratory tract infections, which include the common cold, probiotics have the ability:
    - ☐ increase the duration of the disease
    - ☐ exacerbating pathogenic ailments
    - ☐ Reduce the duration of the disease and alleviate its course
    - ☐ show no ability
    - ☐ I don't know

18. Probiotics are effective in infectious diarrhea in children, by:
    - ☐ only by reducing its duration
    - ☐ only by reducing the number of stools
    - ☐ Shortening its duration and reducing the number of stools
    - ☐ show no effectiveness
    - ☐ I don't know

19. Probiotics play a key role in defense against pathogens in the oral cavity by:
    - ☐ Restoration of the damaged microbiota in the oral cavity and maintenance of its balance
    - ☐ destruction of pathogenic microbiota in the oral cavity
    - ☐ stimulation of proliferation of normal oral microbiota
    - ☐ show no effect
    - ☐ I don't know

20. The microbiota of the digestive system is significantly affected:
    - ☐ Probiotic therapy and a diet rich in fiber
    - ☐ Probiotic therapy and a diet rich in non-food protein
    - ☐ A diet high in saturated fats
    - ☐ A diet rich in simple carbohydrates
    - ☐ I don't know
